# A Macaque's-Eye View of Human Insertions and Deletions: Differences in Mechanisms

**DOI:** 10.1371/journal.pcbi.0030176

**Published:** 2007-09-14

**Authors:** Erika M Kvikstad, Svitlana Tyekucheva, Francesca Chiaromonte, Kateryna D Makova

**Affiliations:** 1 Center for Comparative Genomics and Bioinformatics, Pennsylvania State University, University Park, Pennsylvania, United States of America; 2 Department of Biology, Pennsylvania State University, University Park, Pennsylvania, United States of America; 3 Department of Statistics, Pennsylvania State University, University Park, Pennsylvania, United States of America; University of Chicago, United States of America

## Abstract

Insertions and deletions (indels) cause numerous genetic diseases and lead to pronounced evolutionary differences among genomes. The macaque sequences provide an opportunity to gain insights into the mechanisms generating these mutations on a genome-wide scale by establishing the polarity of indels occurring in the human lineage since its divergence from the chimpanzee. Here we apply novel regression techniques and multiscale analyses to demonstrate an extensive regional indel rate variation stemming from local fluctuations in divergence, GC content, male and female recombination rates, proximity to telomeres, and other genomic factors. We find that both replication and, surprisingly, recombination are significantly associated with the occurrence of small indels. Intriguingly, the relative inputs of replication versus recombination differ between insertions and deletions, thus the two types of mutations are likely guided in part by distinct mechanisms. Namely, insertions are more strongly associated with factors linked to recombination, while deletions are mostly associated with replication-related features. *Indel* as a term misleadingly groups the two types of mutations together by their effect on a sequence alignment. However, here we establish that the correct identification of a small gap as an insertion or a deletion (by use of an outgroup) is crucial to determining its mechanism of origin. In addition to providing novel insights into insertion and deletion mutagenesis, these results will assist in gap penalty modeling and eventually lead to more reliable genomic alignments.

## Author Summary

Insertions and deletions (indels) represent a significant source of evolutionary change. In this manuscript, the authors investigate the patterns of genome-wide rate variation for indels that occurred in the human lineage since its divergence from chimpanzee. Earlier work suggested that insertion and deletion rates are correlated, implying that some genomic factors might affect both types of mutations and thus their patterns of variation across the genome. However, sequences evolving under and without selection were considered together. The present study represents the first attempt to quantify the levels of variation in neutral indel rates in the framework of multiple regression analysis. The finding that insertion versus deletion rates correlate with different genomic features suggests that these two types of mutation are caused in part by distinct molecular mechanisms. This conclusion has direct implications for understanding human genetic diseases, since a large number of them are caused by indels, and contributes to the growing recognition of the importance of fine-scale rearrangement in shaping genome evolution.

## Introduction

Despite the significance of insertions and deletions (indels) for human genetic disease [1] and genome evolution [2–5], the mechanisms of their mutagenesis are not completely understood. Both replication and recombination have been proposed as potential contributors; however, their relative roles in the formation of indels are presently unknown. On the one hand, the importance of replication is supported by the overrepresentation of repeats prone to slipped mispairing [6,7] and of polymerase pause sites in the vicinity of small indels [8]. Male mutation bias observed for indels in rodents is also consistent with their generation by errors in DNA replication [9], because in the germline males undergo more rounds of replication than females [10,11]. On the other hand, while the role of recombination has received much less attention in the literature, its impact may yet be considerable since several motifs known to be associated with recombination are enriched in the proximity of indels [8]. The predominantly maternal origin of indels causing several genetic diseases [12,13], taken together with a higher recombination rate in females than in males [14], also points toward the involvement of recombination. In addition to replication errors and recombination, transcription [15] and aberrations in repair [7] might also cause or facilitate the genesis of indels. Since the rates of all these processes fluctuate across the genome, regional variation in indel rates is expected and its examination should be useful for inferring the mechanisms of insertion and deletion mutations.

Unlike for substitution rates (e.g., [16–18]), a detailed investigation of regional variation in small insertion and deletion rates has not yet been performed. Hardison and colleagues [16] studied regional variation in several different measures of DNA alterations including a rough estimate of the number of bases deleted in either the human or mouse lineages. However, this measure comprised deletions of all sizes; small versus large deletions might be originating by different molecular mechanisms [9]. Additionally, only pairwise relationships between these rough deletion rates and other genomic factors were considered and thus the correlations among these factors were not taken into account. Another study [19] investigated variation in insertion rates of interspersed repeats, yet regional variation in either small insertion or small deletion rates was not examined. Makova et al. [9] analyzed only interchromosomal (and not intrachromosomal) variation in insertion and deletion rates between mouse and rat. Regional variation in human–mouse indel rates was noted by Lunter and colleagues [20]; however, only GC content and chromosomal location were considered to account for this variation and no discrimination was made between insertions and deletions (and they also might be caused by different molecular processes). Finally, additional studies focused on indels situated in genes (e.g., [4,5,8]), which are likely to be affected by selection, leaving indels occurring in neutrally evolving regions largely underanalyzed. Thus, prior to the present study, a systematic analysis of regional variation in small insertion and (separately) deletion rates in neutrally evolving parts of the genome has not been conducted.

Here, we scrutinize patterns of neutral regional variation in rates of small indels across the human genome, and contrast the inferred molecular mechanisms contributing to the generation of insertions versus deletions. We identified small (less than or equal to 30 bp) insertions and (separately) deletions at neutrally evolving (see below) interspersed ancestral repeats (ARs) in the whole-genome human–chimpanzee comparison employing macaque sequences as an outgroup. Next, we applied the methods of multiple regression to rigorously examine genomic factors determining regional variation in rates of these mutations that occurred in the human lineage since the human–chimpanzee divergence.

## Results/Discussion

### Inferring Indels

We established a computational pipeline to detect small indels that occurred in the human lineage since its divergence from chimpanzee, and used the recently sequenced macaque genome [21] as an outgroup to ascertain their polarity in the human–chimpanzee–macaque MULTIZ alignments [22]. Substitution rate matrix and gap penalties were derived for these specific alignments (Materials and Methods, Table S1); gap attraction was found to be minimal (Figure S1). We focused our analyses on indels ≤30 bp because they occur only within (not between) alignment blocks, and thus can be inferred consistently. Also, similar to other studies (e.g., [5]), we gave priority to analyses of human-specific indels because of the higher coverage and quality of the human genome as compared with the chimpanzee genome. To increase the accuracy of indel inference and to minimize false positives, we introduced several levels of filtering (Materials and Methods, Table S2). The analysis was restricted to indels occurring within ARs, which have been widely employed as a model of neutral evolution [16,20]. The resulting dataset consisted of a greater number of human-specific deletions (a total of 237,405) than insertions (a total of 143,904), with size distributions following an exponential decay in both cases (Figure S2), in agreement with other studies [3,8,9]. Insertion and deletion rates, measured here in number of events per base, are conservative because of strict filtering and as a result are lower compared with other studies (e.g., [4]).

### Interchromosomal and Intrachromosomal Variation in Indel Rates

**Figure 1:**
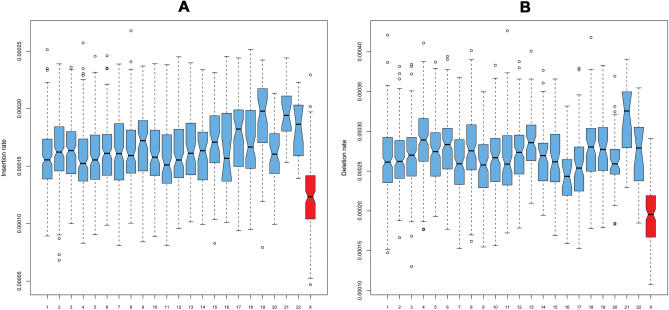
Variation in Human Insertion Rates (A) and Deletion Rates (B) in 1-Mb Windows across the Genome Autosomes (blue) and X chromosome (red) are indicated.

Human-specific insertion and deletion rates are strikingly lower for chromosome X (insertions mean 1.2 × 10^−4^, standard deviation (sd) 3 × 10^−5^; deletions mean 1.9 × 10^−4^, sd 4 × 10^−5^) than for autosomes (insertions mean 1.6 × 10^−4^, sd 3 × 10^−5^; deletions mean 2.7 × 10^−4^, sd 4 × 10^−5^—see also Figure 1). Chromosome Y was excluded from our analysis because a female macaque was sequenced [21]. Significant variation in indel rates is also observed among autosomes (*p* < 3 × 10^−16^, Kruskal-Wallis test over 1-Mb windows; Figure 1; see below).

**Figure 2:**
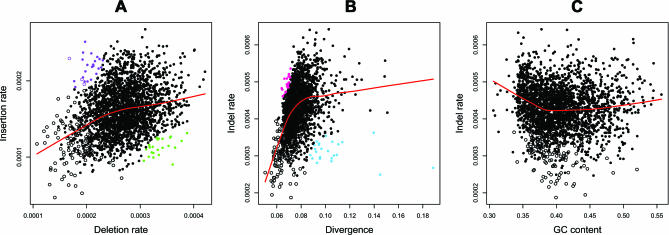
Scatterplots of 1-Mb Windows across the Human Genome (Lowess Smooths Are Superimposed on Each Plot as Visual Aids) (A) Insertion versus deletion rates. (B) Indel rates versus divergence. (C) Indel rates versus GC content. In all three instances, the quadratic fits (*p* < 10^−15^ for (A–C)) explained the data better than linear fits. Empty circles, chromosome X windows; filled circles, autosomal windows; purple circles, windows skewed toward insertions; green circles, windows skewed toward deletions; pink circles, windows skewed toward indels; blue circles, windows skewed toward nucleotide substitutions.

Similar to nucleotide substitutions rates [16–18], small insertion and deletion rates show substantial regional variation (standard deviations computed on 1-Mb windows are 3.2 × 10^−5^ for insertions and 4.5 × 10^−5^ for deletions; Figure S3). Interestingly, a positive association is evident between insertion and deletion rates (Figure 2A), suggesting that at least some of the processes underlying these two types of mutations are shared [8,23].

### Regression Analysis and Choice of a Window Size

**Figure 3:**
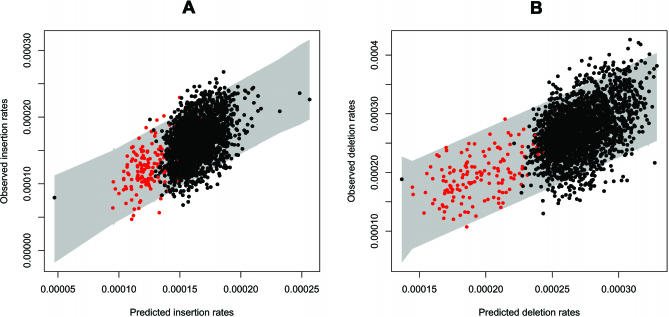
Fitted versus Observed Values for the Final Models for Insertion Rates (A) and Deletion Rates (B) The grey area represents 95% prediction intervals. Data points in red are windows on chromosome X.

To infer the underlying molecular mechanisms contributing to variation in indel rates, we investigated various genomic features as predictors of insertion and deletion rates in 2,568 1-Mb nonoverlapping windows throughout the human genome (Table S3; Materials and Methods) employing a regression analysis. We used the “best subset regression” selection technique to identify linear and quadratic terms for inclusion in our regression models, followed by pruning of terms that were not significant after correction for multiple testing (see Materials and Methods for more details). The two resulting multiple regression models, which explain 32% and 27% of the observed variation in deletion and insertion rates, respectively, are summarized in Table 1 (plots of observed rates versus fitted values from the models, with prediction bands superimposed, are given in Figure 3). For each significant predictor, we calculated its relative contribution to variability explained (RCVE), i.e., the relative amount a predictor contributes to the overall variability explained by the full model in the context of all other predictors. Additionally, we calculated its variance inflation factor—to check for potential estimate deterioration due to multicollinearity (Table 1; Materials and Methods). None of the predictors had variance inflation factor greater than 10; thus, in spite of correlations among predictors (unpublished data), the models are not adversely affected by multicollinearity (Materials and Methods). Thus, we are able to elucidate the individual contributions of each predictor to explaining variability in insertion/deletion rates in spite of correlations between some of them.

**Table 1:**
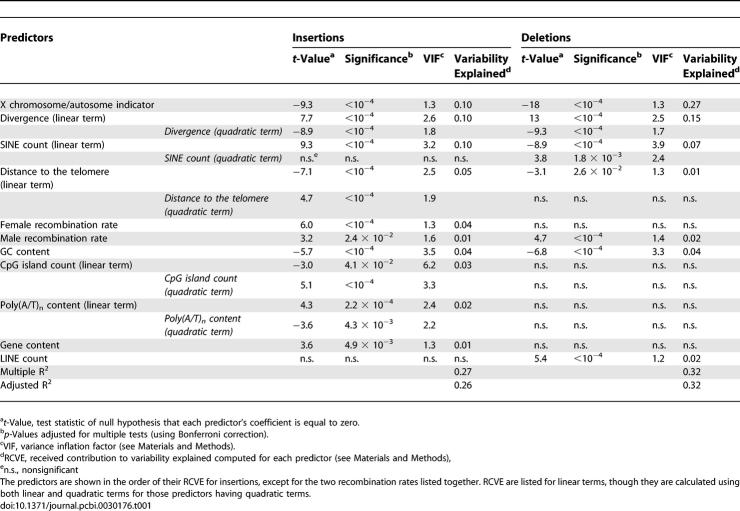
Linear Regression Models for Human-Specific Insertion and Deletion Rates at 1-Mb Genomic Windows

In choosing a window size to perform our main analysis, we struck a balance among various important considerations. Larger windows increase accuracy in the computation of insertion and deletion rates, and thus reduce the error variability carried by these measurements. This fact is reflected in the share of variability explained by our regression models increasing steadily with the window size, for both indels (Table 2). We find no evidence of improved regression performance at sub-Megabase scales (e.g., 100 Kb, 500 Kb), as would be the case if some of the predictors considered in our analysis indeed carried much better explanatory power at such scales. On the other hand, using very large windows (e.g., 5 Mb, 10 Mb) likely causes us to “average out” meaningful variation in rates and predictors, and because of the decreased number of windows, induces overfitting in our regression analysis. 1-Mb windows represent a good compromise in this respect, and are chosen for our main analysis—however, results for different scales are provided in Table 2, and some comparisons are discussed below.

**Table 2:**
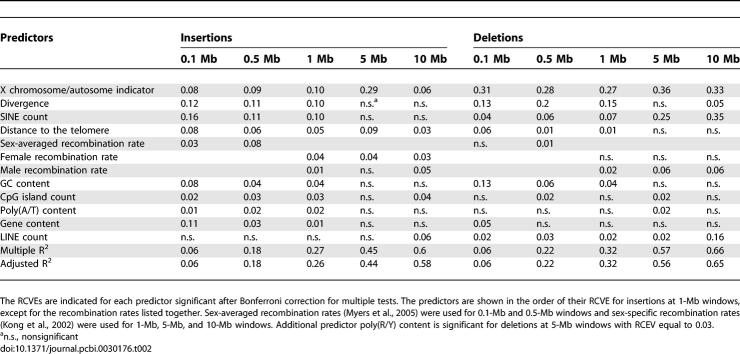
RCVE (see Materials and Methods) for Each Predictor in Linear Regression Models for Human-Specific Insertion and Deletion Rates for Different Window Sizes

The choice of 1-Mb windows is also supported by autocorrelation considerations. Gaffney and Keightley [17] argued that 1 Mb is a “natural” unit of variation for murine substitution rates because the partial autocorrelations among these rates computed in 100-Kb windows are very large at small lags, and rapidly fall to become nonsignificant at lags larger than 10—implying that at scales larger than 1 Mb, similarities among substitution rates can be explained as propagations of similarities at smaller scales [17]. Partial autocorrelation functions computed for human insertion and deletion rates in 100-Kb windows (Figure S4) suggest that the “natural” variation unit may be somewhere between 1 Mb and 3 Mb.

### Common and Different Predictors of Insertion and Deletion Rates

Contrary to expectation [8], the results in Table 1 indicate that some significant predictors are shared by insertions and deletions, while others are not. Factors common to both types of mutations include a categorical variable indicating autosomal versus X-chromosomal location (the X chromosome/autosome indicator), divergence, GC content, male recombination rate, distance to the telomere, and SINE count. In addition, insertion rates correlate with female recombination rates, poly(A/T) content, CpG island counts, and gene content; and deletion rates correlate with LINE counts.

The X chromosome/autosome indicator is one of the top predictors for both insertion and deletion rates. The lower indel rates for chromosome X and the higher rates for autosomes corroborate the importance of replication errors in generating indels [9]; chromosome X spends less time in the male germline, and thus undergoes fewer replications than autosomes [10,11]. The male-to-female mutation rate ratio (alpha; [10]) is ∼7 and ∼17 for insertions and deletions, respectively. These values are, respectively, close to and higher than ∼6, the corresponding ratio in the numbers of male versus female germline cell divisions for human [10], but should be considered preliminary because alpha in closely related species is highly affected by polymorphisms in the ancestral population at the time of speciation [24]. Ancestral polymorphism can lead to an underestimation of divergence and hence obscure estimation of alpha, because the observed divergence between the species pair is in fact a sum of the true fixed divergence and of the ancestral polymorphism [24]. Currently, we cannot properly account for this effect due to the lack of sufficient data for indel polymorphisms; an initial map of human indel polymorphisms indicates equal levels of variation at chromosome X and autosomes [25], which is likely influenced by a deficit of X chromosome traces (a larger study of nucleotide substitution variation found significantly lower polymorphism level on X than on autosomes [26]). Additional indel polymorphism discovery through resequencing (on chromosome X in particular) will be useful in clarifying this issue.

Recombination can also contribute to the observed differences in indel rates between X and autosomes; despite similarity in average recombination rates between these two types of chromosomes in humans [27], the adjusted recombination rate of X is two-thirds that of autosomes (accounting for the fact that X spends only two-thirds of the time in a recombining sex).

By contrast, location on a particular autosome is at best a minor determinant of these rates. Adding chromosomal labels other than X to the regressions leads to either slight or no increase in the total share of explained variability, and to inconsistent results at various scales that are difficult to interpret biologically (specifically, different autosomes appear significant at different window sizes; Table S6). Moreover, the inclusion of autosomal labels does not alter results relative to other predictors (Table S6). All further discussion is therefore based on the regressions presented in Table 1, which do not include autosomal labels.

A significant positive association between deletion or insertion rates and divergence (Table 1, Figure 2B), also reported elsewhere [4,16,23], supports the claim that some regions of the genome possess elevated mutability for several types of mutations [16]. The correlation between indel and nucleotide substitution rates can also be interpreted as evidence for the importance of replication in mutagenesis of indels; it is accepted that replication errors cause a substantial portion of nucleotide substitutions [11,28]. Note that divergence is a stronger predictor of deletion as compared with insertion rates.

The association between GC content and both insertion and deletion rates again emphasizes the role of replication in generating indels. Indeed, the curvilinear relationship that is observed by plotting indel rates against GC content (Figure 2C) supports the origin of indels via replication slippage; GC-poor and GC-rich regions have a high occurrence of mononucleotide runs leading to increased slippage, and as a result to elevated indel rates [20]. Additionally, GC content is known to correlate with replication timing [29].

While both insertions and deletions are associated with male recombination rates, insertions are additionally and more strongly associated with female recombination rates (Table 1). These observations imply that more insertions than deletions are linked to recombination, and that recombination-mediated insertions might occur preferentially during female meiosis. There are established differences between the sexes in terms of meiosis and recombination that could contribute to these observations. For instance, compared with males, females display recombination rates that are higher on average and have a distinct intrachromosomal distribution: female rates are higher at centromeres, and not as elevated at telomeres [14,30]. Additionally, female meiosis is known to be more error-prone and to have 2-fold longer synaptonemal complexes than male meiosis [31]. Notably, in mouse, knockouts of several proteins important for double-strand break formation and repair lead to more pronounced defects for spermatogenesis than oogenesis [31]. Thus, our results are consistent with mechanistic differences between the two types of mutations that might be caused by such sexual dimorphism in recombination rates and/or in mammalian meiosis.

Puzzlingly, similar to nucleotide substitutions [32], both insertion and deletion rates increase near the telomeres, with a stronger effect for insertions than for deletions (Table 1). Several other predictors in the model co-vary with distance to telomeres—e.g., recombination rate [27] and GC-content [33]—but the multiple regression approach allows us to evaluate each predictor, factoring out overlapping effects of other predictors included in the model (see above). Thus, we have evidence that yet-unidentified factors may contribute to higher rates of both small indels and nucleotide substitutions near the telomeres [18,32]. Chromosome ends are known to possess special properties that provide clues to explaining elevated mutation rates in their vicinity. For instance, some distal regions do not exhibit the association between early replication and open chromatin that is observed in other regions of the genome [34]. Moreover, subtelomeric regions are enriched in sites undergoing meiotic nonhomologous end-joining, one of the major mechanisms of double-strand break repair in mammals [35]. These regions also have elevated rates of mitotic sister chromatid exchange, indicating that distal regions of the chromosome are subject to double-stranded breaks and/or repair at much greater frequencies than internal regions [36]. Faulty repair can potentially lead to indel formation.

The incidence of SINEs appears to be strongly associated with both insertion and deletion rates, albeit in opposite ways (Table 1). A positive association between insertion rates and SINE counts might reflect the prominent role that SINEs play in promoting non-allelic homologous recombination (particularly between Alus [37]), and is consistent with the importance of recombination for this type of mutation (see above). Conversely, deletion rates display a strong negative association with SINE counts. Nucleotide substitution rates are also known to correlate negatively with the occurrence of SINEs [19]. The exact explanation for this phenomenon is unclear, however; SINEs are known to accumulate in different (usually GC-rich) portions of the genome as compared with other repeats [38]. Similarly to nucleotide substitutions [19], deletions (but not insertions) are positively associated with occurrence of LINEs (Table 1).

Insertion rates rise with increasing content of poly(A/T) runs (Table 1), which have been connected with high recombination rates [14] and are locally enriched near indel sites [6,8]. Because of their repetitive nature, poly(A/T) runs are also expected to be frequent sites of replication slippage events [7]. Furthermore, insertion rates are correlated with gene content and CpG islands (Table 1), although these predictors explain only a small fraction of the overall variability.

Fitting regressions for different window sizes allows us to investigate the scales at which each genomic feature is the most highly connected to indel rates (Table 2). For instance, divergence, which is claimed to “naturally” vary at ∼1 Mb scale [17], has higher RCVE at smaller scales. Similarly, GC content, known to vary even more locally [39], loses its significance at larger window sizes. In contrast, the share of variability in deletion rates explained by SINEs increases with window size. The predictive power of the X/autosomal indicator and recombination rates (sex-averaged, male- or female-specific), though fluctuating in magnitude, remains significant at almost all scales considered in Table 2 (recombination rates are not significant at 0.1-Mb windows for deletions).

### Analysis of Human-Specific 1-bp Indels and Autosomal-Only Data

In addition to considering different scales, we performed regressions (at the 1-Mb scale) for 1-bp human-specific indels, which constitute roughly 50% of our total dataset (Table S4), and (separately) for autosomal windows only (Table S5). The results of these analyses were largely consistent with our main findings. However, the total share of explained variability was lower than for the regressions summarized in Table 1. The lower total share of variability explained for the 1-bp analysis can be attributed to the fact that the smaller number of indel events available in each window decreases the accuracy in the calculation of the rates.

Despite the significance of the X chromosome/autosome indicator (Table 1), an analysis of autosomal-only windows confirms the differences in the relative contributions of the remaining genomic factors contributing to variation in indel rates (Table S5). Notably, the significance of the predictors is very similar to the genome-wide analysis reported in Table 1. The decrease in total share of variability explained can be attributed to the importance of the observed differences in indel rates between X and autosomes.

### Windows with Extremely Different Insertion versus Deletion Rates

To further explore differences in the biological mechanisms contributing to indels, we identified and analyzed 1-Mb windows having extremely different insertion versus deletion rates. We selected 25 windows skewed toward insertions and 25 windows skewed toward deletions, each group constituting ∼1% of our original dataset (Materials and Methods; Figure 2A). Contrasting such windows allows us to highlight factors more important for one type of mutation versus the other. This analysis confirmed our main results (Table 1). For instance, the incidence of SINEs is significantly higher in windows skewed toward insertions than in ones skewed toward deletions (*p* < 0.0001, one-tailed randomization test comparing two medians; Materials and Methods), verifying the opposite effect of SINE count on the two types of mutations. Female recombination rates, found to be positively associated with insertion rates, are significantly higher in windows skewed toward insertions than in windows skewed toward deletions (*p* = 0.0051).

Similarly, inspection of windows with extremely different indel rate versus divergence allows us to identify genomic factors having stronger association with either indels or nucleotide substitutions (Figure 2B; Materials and Methods). For instance, windows skewed toward nucleotide substitutions are significantly closer to telomeres than windows skewed toward indels (*p* = 0.0033), suggesting a greater importance of distance to telomere for nucleotide substitutions than indels. On the contrary, LINE counts are significantly higher in windows skewed toward indels than in windows skewed toward substitutions (*p* = 0.0116), suggesting a stronger association with deletions than divergence (insertions are likely unaffected by LINE count; Table 1).

### Indel Rates and Most Conserved Elements

**Figure 4:**
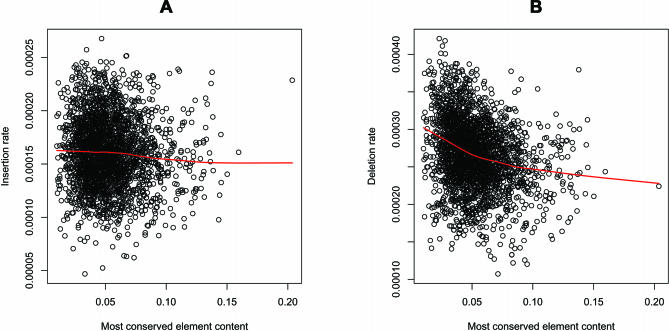
Insertion and Deletion Rate Variation as a Function of the Fraction of the Window in the Most Conserved Elements Lowess smooths are surperimposed on each plot as visualization aids.

In a separate analysis, we investigated relationships between insertion or deletion rates and the proportion of a window occupied by so-called most conserved elements (i.e., content). Many of these elements are likely to be functional and include protein-coding exons, other transcribed regions, and conserved noncoding sequences potentially important for gene regulation and other cellular processes [40]. Interestingly, we found a sizeable negative correlation (Pearson's correlation coefficient *r* = −0.26, *p* < 0.0001; Figure 4) between deletion rates and content of most conserved elements (the correlation for insertion rates was much weaker; *r* = −0.053, *p* = 0.0070; Figure 4). In agreement with this, the content of most conserved elements was significantly lower in windows skewed toward deletions than in windows skewed toward insertions (*p* < 0.0409). The indel rates studied here are estimated at ARs, known to have very little overlap with most conserved elements [40]. In spite of this, these results suggest, intriguingly, that regions of the genome dense in highly conserved (and likely functional) elements evolved to have low deletion rates, while still tolerating a certain amount of insertions. At the same time, we have evidence that these regions might tolerate more indels than substitutions—the content of most conserved elements is significantly lower in windows skewed toward substitutions than in windows skewed toward indels (*p* = 0.0004).

### Conclusions

The above discussion of genomic factors suggestive of similarities and differences in the mutagenesis of insertions and deletions leads to the following conclusions. First, our regression analyses are consistent with the importance of replication for generating both indels [7,9]. Indeed, many shared significant predictors either alter the probability of replication errors (e.g., GC content and the X/autosomal indicator, although the latter can also be affected by recombination rate differences) or are caused by them (e.g., divergence). Next, while slipped mispairing has received most of the attention as a model for small indel mutagenesis, our results indicate that replication alone cannot account for all indel events. In contrast to analyses focusing on genes and emphasizing the role of replication [8], we find that recombination rates are significant predictors for both insertions and deletion rates in the neutrally evolving regions of the genome, contradicting a recent study [41].

Finally, the differences between genomic factors significantly associated with insertions and deletions suggest that the relative contributions of replication versus recombination are unequal for these two types of mutations. The trends we observe for deletions are closer to those reported for nucleotide substitutions in other studies (e.g., positive association with male recombination rates and negative association with SINE occurrence [19]). In agreement with this, divergence is a stronger predictor for deletions than for insertions (Table 1). Since many nucleotide substitutions result from errors in DNA replication [11,28], it is plausible that a large fraction of deletions are caused by replication-associated mechanisms as well. However, some deletions are probably caused by recombination as suggested by the significance of male recombination rates in our regression analysis, as well as by another study [42]. In stark contrast, female recombination rate and other recombination-related forces (e.g., positive association with SINE frequency) are strongly connected to insertions. Thus, even though these patterns are difficult to quantify as a whole, our results suggest that replication-related factors are mostly important for deletions, while recombination-related effects are more pronounced for insertions. This is consistent with observations of unequal rates of insertions versus deletions, and distinct motifs for insertion and deletion hotspots [6,8].

Differentiating between insertions and deletions using the macaque sequence as an outgroup has implied that nontrivial mechanistic differences exist between the two types of mutations. The importance of recombination and replication to indel formation conveyed here warrants evaluation in future studies. Conceivably, such studies will also allow us to discriminate between the roles of replication and repair. Our in-depth investigation of neutrally evolving indels provides important insights into indel mutagenesis, with its implications for understanding human genetic diseases. In addition, it will aid in the development of better gap modeling techniques, which are crucial for improving alignment methodology and thus for inferences on genome evolution.

## Materials and Methods

### Inferring indels.

Alignment methods and parameters are critical for the identification of indels. Here we use the human–chimpanzee–macaque (hg18–panTro2–rheMac2) three-way genome alignments that were produced by the MULTIZ algorithm [22] and are available at the University of California Santa Cruz Genome Browser (UCSC) (http://genome.ucsc.edu). This BLASTZ-based local alignment tool was used to generate alignments of other mammalian genomes, and thus allows a comparison of our results with those of other studies, which employ comparisons of more diverged genomes (e.g., [9]). Correct placement of gaps in an alignment depends on the interplay between substitution rate matrix and gap penalties. Our pilot analysis indicated that the default MULTIZ matrix and gap penalties derived from human–mouse alignments are inappropriate for human–chimpanzee alignments. We therefore derived new parameters (Table S1): the substitution rate matrix was obtained by a probabilistic scoring scheme [43], and the corresponding matrix-specific gap penalties were obtained by empirical testing, similar to other studies [44]. Although a rigorous statistical procedure to derive alignment gap penalties is currently unavailable, developing such procedure represents an active area of research [45–47].

If indel events are independent, intergap distances are expected to follow a geometric distribution [20]. However, an under-representation of short intergap distances (<20 bp) due to gap attraction has been noted for human–mouse alignments [20]. For alignments used in the present study, gap attraction is minimal and only occurs for intergap distances smaller than 4 bp (Figure S1). Consequently, we do not adjust gaps manually.

MAF-formatted alignment blocks were restricted to human coordinates of ARs using Galaxy [48]. ARs were defined as RepeatMasker [49] annotations of DNA elements, LTRs, LINEs, and SINEs, excluding elements active since the human–macaque divergence time, namely L1PA1-A7, L1HS, and AluY [38,50,51].

Custom PERL scripts (available upon request) were developed for the computational pipeline to identify and filter indels. A *human-specific deletion* was identified as a gap of one or more consecutive columns within a MAF local alignment block, covered by a nucleotide base at the orthologous position in both the chimpanzee and macaque genomes. Conversely, a *human-specific* insertion was defined as one or more consecutive nucleotides in human covered by gaps in both the chimpanzee and macaque genomes.

Filtering of putative indels was further performed to remove potential false positives (Table S2). *First*, we excluded indels occurring in overlapping blocks of local alignments to avoid scoring the same loci more than once, which might result in indel rate inflation. This effectively removed most of the indels located in segmental duplications. In the final dataset, only ∼1% of indels overlapped with coordinates of segmental duplications as annotated by the UCSC Genome Browser (as a comparison, ∼5% of the genome consists of segmentally duplicated regions; [52]). *Second*, microsatellites, simple repeats, and regions of low complexity as annotated by RepeatMasker for each of the three genomes were eliminated because they are usually difficult to sequence, assemble, and align accurately. *Third*, we required a minimum of three bases flanking each side of a gap to have a quality Phred score of 20 or higher in both the chimpanzee and macaque draft genomes. Indels violating this criterion were excluded. *Fourth*, gaps occurring at orthologous locations but having unequal length in different species were filtered out because such instances represent either sequence errors or multiple indel events [53,54]. Insertion and deletion rates were then calculated as the number of events per aligned (ungapped) human base in ARs covered by three-way alignments, correcting the denominator for nucleotides excluded due to filtering.

### Windows and predictors for multiple regression analyses.

To investigate regional variation in indel rates, we divided the human genome (hg18) into nonoverlapping windows and estimated counts or content (fraction of bases of the window) for various genomic features to create a set of potential predictors (Table S3). The features (based on the hg18 annotations in the UCSC Genome Browser) were calculated at the level of 100-kb windows, and aggregated as needed when considering larger windows.

The calculation of recombination rates is an exception to this rule, since different sources were used for different window sizes. For 1-, 5- and 10-Mb windows, sex-specific recombination rates were obtained from the UCSC Genome Browser deCODE data track [14] for build hg18. For this track, marker DNA is aligned to each new assembly, thus the coordinates are taken into account when determining the corresponding physical distance (the UCSC Genome Bioinformatics Group, personal communication). For 0.1- and 0.5-Mb windows, the computationally predicted recombination rates obtained from linkage disequilibrium analysis of human SNP data [55] were utilized, as sex-specific rates are not available at these scales.

Human–macaque divergence was used instead of human–chimpanzee divergence because of a strong effect of ancient polymorphisms on the latter [24], and was calculated at ARs using a REV model, as implemented in the *baseml* module of PAML [56].

In addition to various quantitative predictors obtained as counts and frequencies, we considered an indicator variable which labels each window as belonging to X (“1”) or autosomes (“0”)—this is also listed in Table S3. Some of our fits also included labels for specific autosomes (see Table S6). The male-to-female mutation rate ratio, or alpha, was calculated according to [57].

Windows were excluded from the analysis at two stages of filtering; first, if they lacked data due to low sequencing coverage (“N” content >50% of the window) or if they lacked sufficient aligned AR coverage (<20% of the window). Second, additional windows were excluded due to lack of recombination data and/or human–macaque divergence estimates. As the X chromosome is unique in having distinct ordered physical and evolutionary blocks or “strata” (based on divergence from Y; [58]), windows located in pseudoautosomal and evolutionary stratum 5 regions of this chromosome were also excluded as they did not evolve as truly X-like over the evolutionary time examined. For instance, for 1-Mb windows, the initial total was 3,010, the total after the first filtering 2,614, and the total after the second filtering 2,568.

### Model selection and fitting.

All computations were conducted using the R statistical package [59]. Standard regression diagnostics were used to identify and remove outliers (Cook's distances, standardized residuals greater than 3 or smaller than −3), assess goodness of fit for each model (plots of residuals versus fits, normal probability plots), and evaluate predictors (added variable plots, *t*-tests and general linear F-tests, partial R^2^ and similar measures, variance inflation factors (VIF)); see [60] for details on these diagnostic techniques. In addition, the best subset regression selection procedure was employed to identify subsets of linear and quadratic terms to include in final regression models.

For both insertion and deletion rates (separately), model selection was performed at the 1-Mb scale (and similarly at other scales), with the following approach. We started with the pool of predictors in Table S3, and formed an overall set of terms comprising all linear and quadratic terms in the quantitative predictors and the X/autosomal indicator. Including squared terms allowed us to account for possible curvatures in the relationship with the response. This overall set of terms was subjected to a “best subset” selection procedure, which identified subsets with smallest Mallow's Cp value [60]. Similar to an adjusted R^2^, Mallow's Cp selects subsets based on a balance between small mean square error (MSE) for the corresponding regressions, and parsimony (small number of terms).

Next, the regressions corresponding to the best subsets for insertion and deletion rates were further “pruned,” eliminating terms whose coefficients were not significant after a Bonferroni correction [60] or which carried large VIFs (see below). This led to the final models summarized in Table 1. Adding autosomal labels as indicators to these regressions was also considered (Table S6), but produced only minor improvements in terms of R^2^, with inconsistency in the results when refitting the models for different window sizes (Table S6).

While many of the quantitative predictors are correlated, our regression fits for both indels are not adversely affected by multicollinearity, as shown by the relatively low values of the VIFs in Table 1. VIFs are commonly used to diagnose multicollinearity. The VIF of a term in a regression measures how much the variance of its estimated coefficient increases relative to what it would have been if all predictors were orthogonal [60]. High values of VIF (greater than ten) indicate that the accuracy of the regression coefficient estimates is eroded by collinearity (intuitively, that the least square solution is “less stable” because of the linear interdependencies among the predictors). VIF value of 1 means orthogonal (uncorrelated) predictors. All predictors from our final regressions reported in Tables 1, 2, and S4–S6 had VIFs lower than 10.

To assess the contribution of each individual predictor to the explanation of the total variability in the response, we use RCVE:

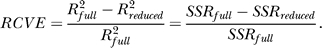



Here 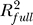 and *SSR_full_* are the R^2^ (share of variability explained) and the regression sum of squares of the full model (includes all significant terms), while 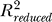 and *SSR_reduced_* are the corresponding quantities for the model obtained from the full model dropping all terms involving the predictor of interest (i.e., both linear and quadratic terms where applicable). The RCVE expresses the relative contribution of a predictor in the context of all other predictors included in the full model. Therefore, if the predictors are correlated, because of “overlaps” in the contribution, the numerators of the RCVEs do not add up to 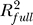 , and the RCVEs do not add up to 1. The RCVE is a close relative of a more commonly used measure called partial R^2^—the formula for the latter has *SSE_reduced_* (the error sum of squares for the reduced model) instead of *SSR_full_*, at the denominator. We find the RCVE to be more intuitive than the partial R^2^ because it uses the same denominator for all predictors in the same model. We evaluated the predictors in our models using the standard partial R^2^, with very similar results and consistent conclusions (unpublished data).

We also checked whether residuals from our final regression models presented troublesome spatial autocorrelations among adjacent windows on each chromosome. Diagnostic plots of the residuals' partial autocorrelation function (PACF) for various lags (here lags are measured in number of adjacent 1-Mb windows) showed no substantial evidence against the assumption of independent errors on which the regression fits rely (autocorrelation parameter values <0.2 do not violate the assumption of independent error terms); moreover, the partial autocorrelation in residuals drops substantially compared with the partial autocorrelation in the response (unpublished data).

### Identification of windows with extremely different rates.

To further explore differences between indels, as well as substitutions, we considered a number of features (some chosen among the predictors in our regression analysis, and some novel—e.g., most conserved element content obtained from the UCSC Genome browser [40]), and compared them between groups of windows presenting very extreme behaviors in terms of these mutations. The groups were identified as follows.

We ranked all windows used in the 1-Mb regression analysis according to insertion and deletion rates separately. Next, we computed the difference between each window's ranks in terms of insertion and deletion rates, and selected windows in the ∼1% left and right tails of the distribution of rank differences. These two groups (25 1-Mb windows each) represent genomic locations extremely skewed toward deletions (versus insertions) and toward insertions (versus deletions), respectively. Note that this rank analysis is completely nonparametric and robust to the nature of the relationship between the two mutation types.

Median values of some regression predictors and other variables (e.g., fraction of a window covered by most conserved elements) were calculated for the two groups. To test whether differences in medians between the two groups were significant, we used a randomization procedure. We randomly sampled (without replacement) two groups of 25 windows each, and computed the differences in medians between them for all variables considered. Repeating this 10,000 times allowed us to construct empirical null distributions for each difference in medians for variables of interest, and thus empirical *p-*values.

The same approach was used to identify windows extremely skewed toward indels (versus substitutions) and toward substitutions (versus indels), and to test for differences in medians for various variables between these two groups. The windows analyzed in this section were randomly distributed among and within chromosomes (i.e., did not cluster to specific regions in the genome).

## Supporting Information

Figure S1 Distribution of Intergap Distances Plot (log_10_ scale) of intergap distance counts in human–chimpanzee–macaque alignments calculated in ARs after filtering. The data are shown for chromosome 1 only and are representative of the genome-wide distribution. The distribution follows closely the predicted geometric shape, with deviation only in the range of ≤4 bp.
(2.2 MB PDF)

Figure S2 Relative Size Distribution of Human Insertions (White) and Deletions (Black) (1.6 MB PDF)

Figure S3 Variation among Human Insertion and Deletion Rates in 1-Mb Windows In the box plots, edges correspond to quartiles and vertical dashed lines to the range. Notches represent standard deviations of the median. Nonoverlapping notches are evidence that the two medians differ.
(2.4 MB PDF)

Figure S4 Partial Autocorrelation Functions for Insertion Rates (A) and Deletion Rates (B) in 100-kb Windows (1.5 MB PDF)

Table S1 BLASTZ Human–Chimpanzee Alignment Parameters (37 KB DOC)

Table S2 The Numbers of Human Indels Filtered at Each Step (26 KB DOC)

Table S3 Predictors Used in the Regression Analysis Features were calculated as observed counts or contents (fraction of bases) in a window.
(39 KB DOC)

Table S4 Linear Regression Models for 1-bp Human-Specific Insertion and Deletion Rates Calculated at 1-Mb Windows (91 KB DOC)

Table S5 Linear Regression Models for Human-Specific Insertion and Deletion Rates Calculated in 1-Mb Windows, for Autosomal Data Only (83 KB DOC)

Table S6 Linear Regression Models for Human-Specific Insertion and Deletion Rates for Different Window Sizes after Including Chromosomal Labels The RCVE is indicated for each predictor significant after Bonferroni correction for multiple tests. For 5-Mb windows, no autosomal labels were identified as significant predictors for both indels. For 10-Mb windows, only insertions had significant autosomal labels.
(71 KB DOC)

## Abbreviations

AR: ancestral repeat
indels: insertions and deletions
RCVE: relative contribution to variability explained
VIF: variance inflation factors
