# Supplementary material for: A Macaque's-Eye View of Human Insertions and Deletions: Differences in Mechanisms
Source: PLoS Comput Biol. 2007 Sep 14;3(9):e176. doi: 10.1371/journal.pcbi.0030176 (PMC1976337; doi:10.1371/journal.pcbi.0030176)
Supplement: Supplementary file 2 [file pcbi.0030176.sg002.pdf]

S2

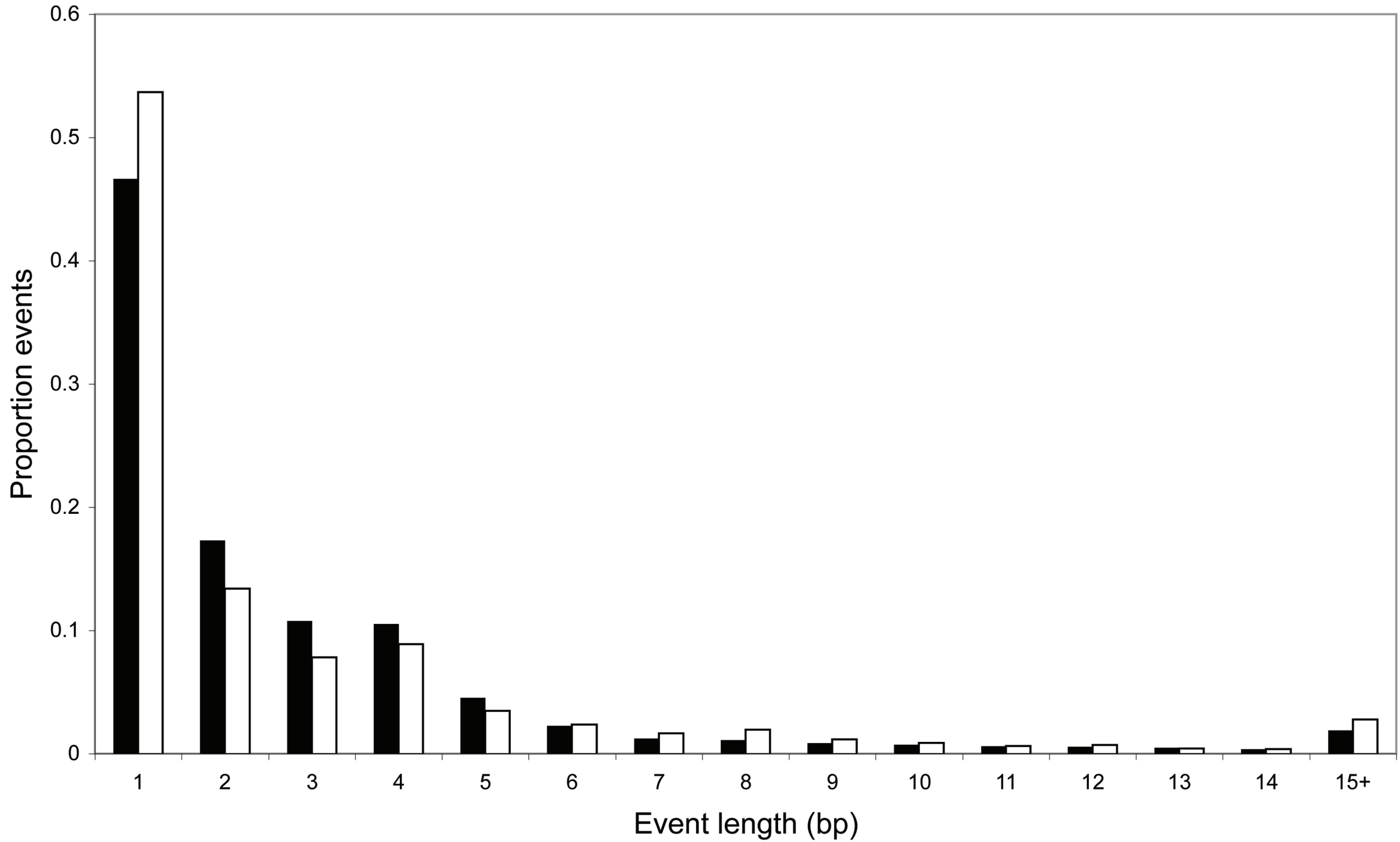
0.6
0.5
0.4
0.3
0.2
0.1
0
Proportion events
1
2
3
4
5
6
7
8
9
10
11
12
13
14
15+
Event length (bp)
