# Supplementary material for: A Macaque's-Eye View of Human Insertions and Deletions: Differences in Mechanisms
Source: PLoS Comput Biol. 2007 Sep 14;3(9):e176. doi: 10.1371/journal.pcbi.0030176 (PMC1976337; doi:10.1371/journal.pcbi.0030176)
Supplement: Supplementary file 3 [file pcbi.0030176.sg003.pdf]

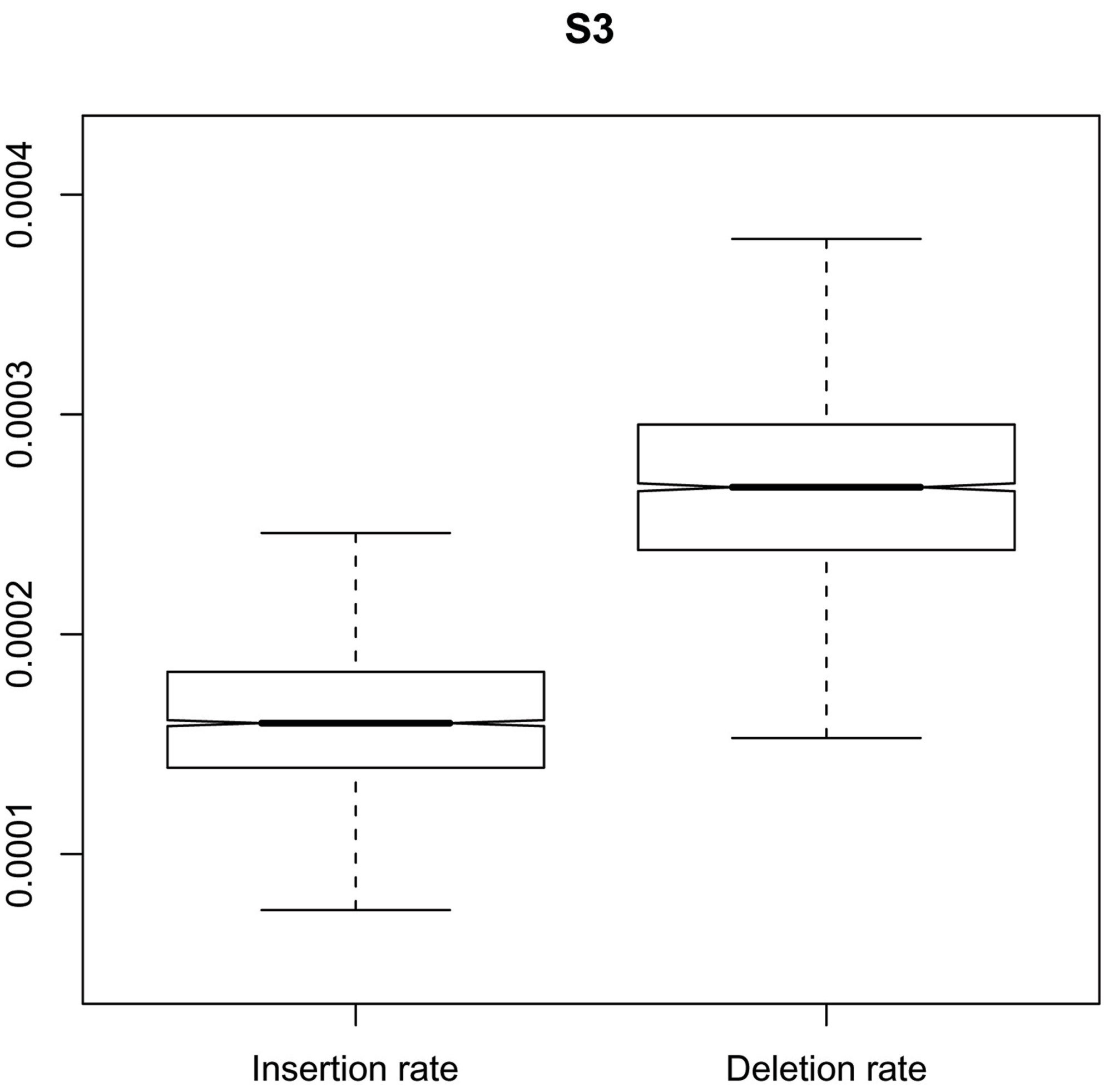
S3
0.0001
0.0002
0.0003
0.0004
Insertion rate
Deletion rate
