# Supplementary material for: A Macaque's-Eye View of Human Insertions and Deletions: Differences in Mechanisms
Source: PLoS Comput Biol. 2007 Sep 14;3(9):e176. doi: 10.1371/journal.pcbi.0030176 (PMC1976337; doi:10.1371/journal.pcbi.0030176)
Supplement: Supplementary file 4 [file pcbi.0030176.sg004.pdf]

S4 A

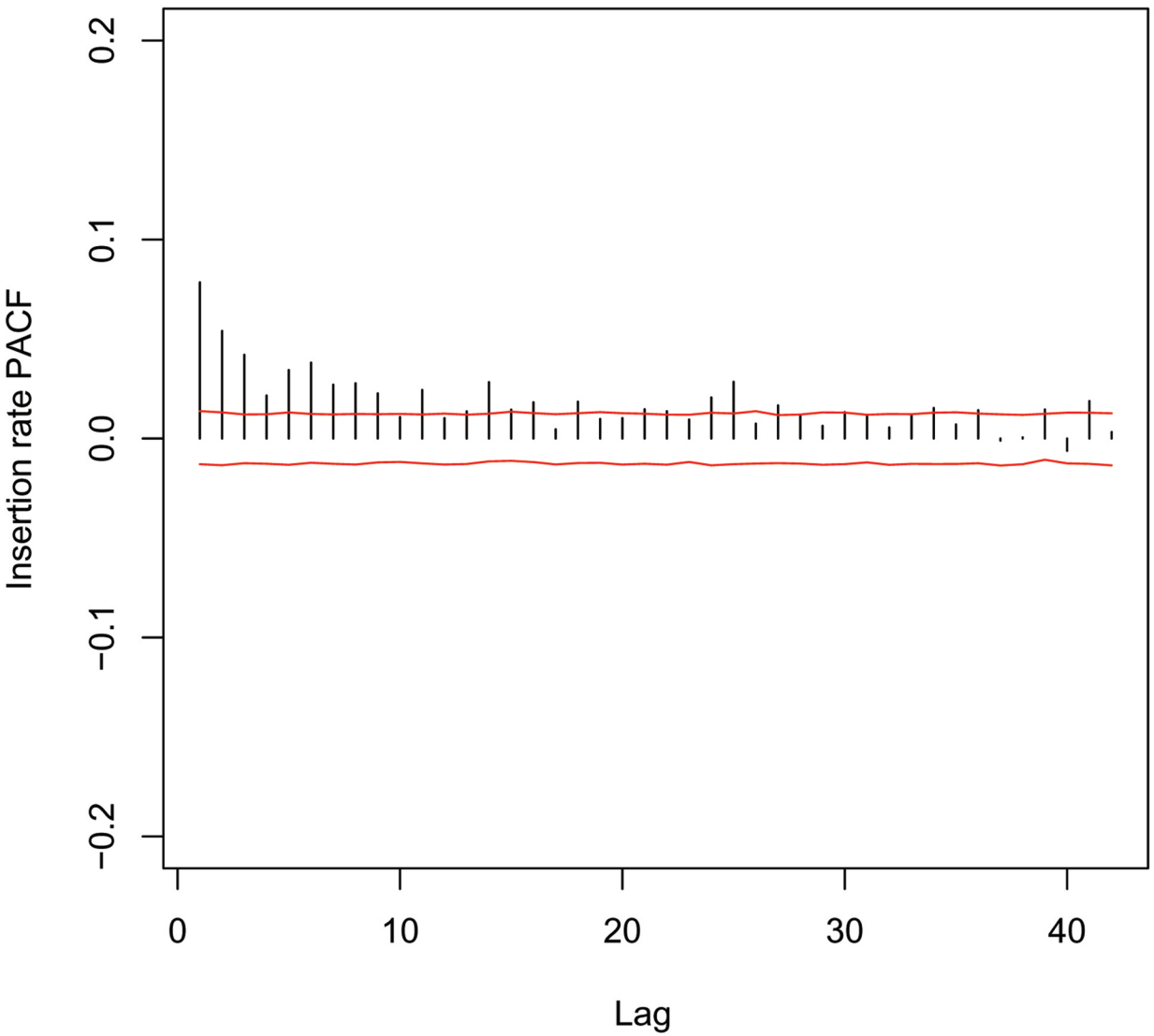

Insertion rate PACF
0.2
0.1
0.0
−0.1
−0.2
0
10
20
30
40
Lag

S4B

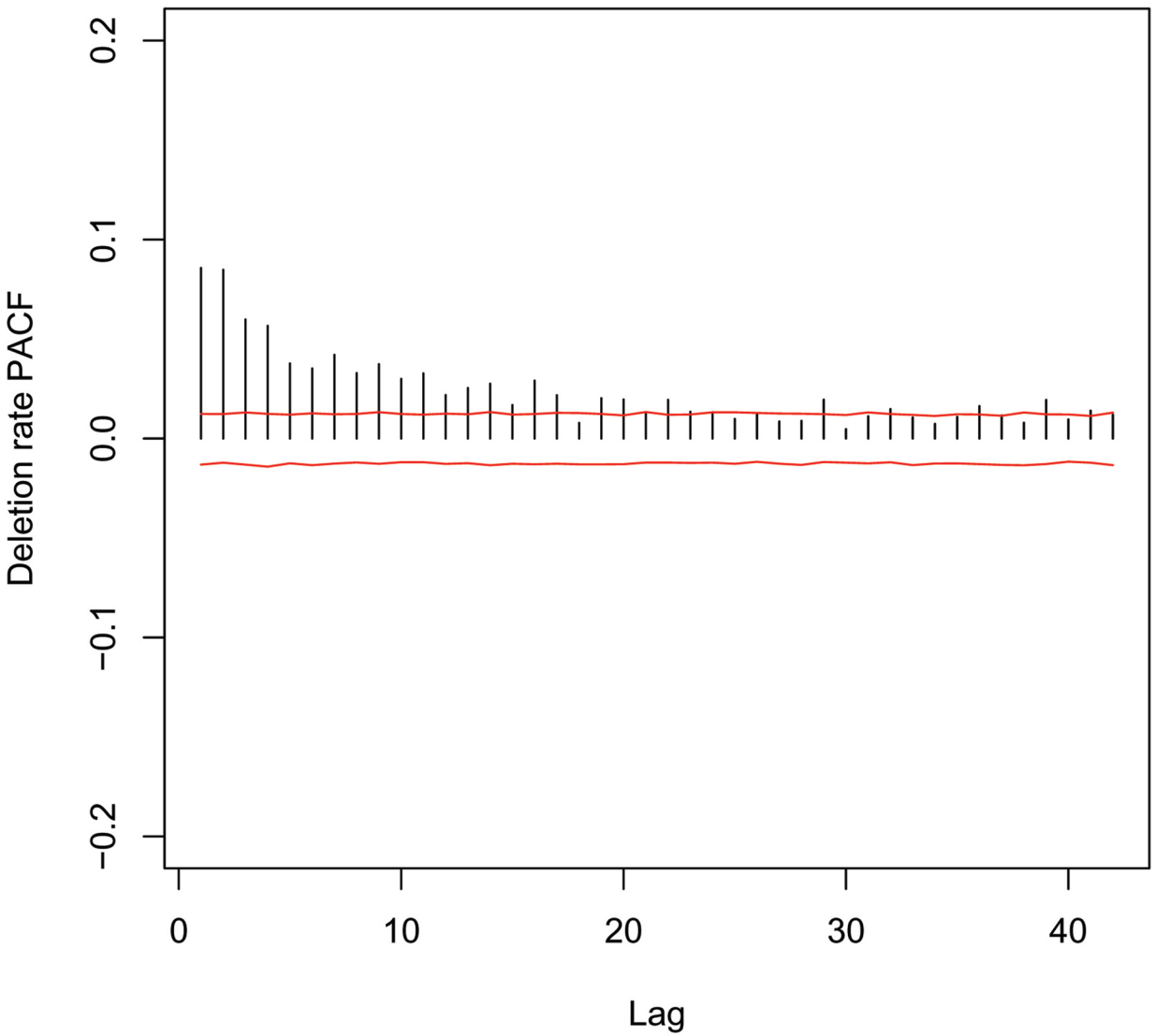

Deletion rate PACF
0.2
0.1
0.0
−0.1
−0.2
0
10
20
30
40
Lag
