# Supplementary material for: A Macaque's-Eye View of Human Insertions and Deletions: Differences in Mechanisms
Source: PLoS Comput Biol. 2007 Sep 14;3(9):e176. doi: 10.1371/journal.pcbi.0030176 (PMC1976337; doi:10.1371/journal.pcbi.0030176)
Supplement: Supplementary file 5 [file pcbi.0030176.st001.doc]

| **A. Default BLASTZ parameters** | | | | **B. Human-chimpanzee parameters** | | | |
| --- | --- | --- | --- | --- | --- | --- | --- |
| A | C | G | T | A | C | G | T |
| 91 | -114 | -31 | -123 | 90 | -330 | -236 | -356 |
| -114 | 100 | -125 | -31 | -330 | 100 | -318 | -236 |
| -31 | -125 | 100 | -114 | -236 | -318 | 100 | -330 |
| -123 | -31 | -114 | 91 | -356 | -236 | -330 | 90 |
|  |  |  |  |  |  |  |  |
| O=400, | E=30, | Y=9400 |  | O=600, | E=150, | Y=15000 |  |

O : gap open penalty

E : gap extension

Y: X-drop parameter for gapped extension
