# Supplementary material for: A Macaque's-Eye View of Human Insertions and Deletions: Differences in Mechanisms
Source: PLoS Comput Biol. 2007 Sep 14;3(9):e176. doi: 10.1371/journal.pcbi.0030176 (PMC1976337; doi:10.1371/journal.pcbi.0030176)
Supplement: Supplementary file 6 [file pcbi.0030176.st002.doc]

| Filter | The number (and %) of indels |
| --- | --- |
| Filter 1: overlapping blocks | 18,898 (3%) |
| Filter 2: low complexity | 22,826 (4%) |
| Filter 3: low quality | 20,010 (3%) |
| Filter 4: overlapping events | 203,608 (31%) |
| Total filtered out | 265,342 (41%) |
| Total after filtering | 381,309 |
| Total | 646,651 |
