# Supplementary material for: A Macaque's-Eye View of Human Insertions and Deletions: Differences in Mechanisms
Source: PLoS Comput Biol. 2007 Sep 14;3(9):e176. doi: 10.1371/journal.pcbi.0030176 (PMC1976337; doi:10.1371/journal.pcbi.0030176)
Supplement: Supplementary file 7 [file pcbi.0030176.st003.doc]

| Feature | Calculation |
| --- | --- |
| GC content | fraction of bases per window |
| CpG island count | counts per window |
| Gene content^a^ | fraction of bases per window |
| SINE count | counts per window |
| LINE count | counts per window |
| Human-macaque divergence | estimated in ARs with REV |
| Distance to the telomere | from midpoint coordinate of the window |
| Poly(A/T)_n_ content | fraction of bases per window (n>=4) |
| Poly(R/Y)_n_ content | fraction of bases per window (n>=5) |
| Female recombination rate | from Kong et al. (2002) |
| Male recombination rate | from Kong et al. (2002) |
| Computational recombination rate | from Myers et al. (2005) |
| The X chromosome/autosome indicator | “0” for autosomes; “1” for X |

^a^Similar to Hellmann et al. (2005), gene content was calculated as fraction of bases per window located between CDS annotated start and stop coordinates of known genes.
