# Supplementary material for: A Macaque's-Eye View of Human Insertions and Deletions: Differences in Mechanisms
Source: PLoS Comput Biol. 2007 Sep 14;3(9):e176. doi: 10.1371/journal.pcbi.0030176 (PMC1976337; doi:10.1371/journal.pcbi.0030176)
Supplement: Supplementary file 8 [file pcbi.0030176.st004.doc]

A. Insertions

| Predictors | *t* value^a^ | Significance^b^ | VIF^c^ | Variability Explained^d^ |
| --- | --- | --- | --- | --- |
| X chromosome/autosome indicator | -12 | < 10^-4^ | 1.0 | 0.46 |
| Divergence | n.s.^e^ | n.s. | n.s. | n.s. |
| SINE count | n.s. | n.s. | n.s. | n.s. |
| Distance to the telomere (linear term) | -7.9 | < 10^-4^ | 1.8 | 0.20 |
| *Distance to the telomere (quadratic term)* | 4.7 | < 10^-4^ | 1.5 |  |
| Female recombination rate (linear term) | 5.2 | < 10^-4^ | 1.9 | 0.09 |
| *Female recombination rate (quadratic term)* | -3.3 | 1.1x10^-2^ | 1.6 |  |
| Male recombination rate | n.s. | n.s. | n.s. | n.s. |
| GC content (linear term) | -2.9 | 4.92x10^-2^ | 7.4 | 0.03 |
| *GC content (quadratic term)* | n.s. | n.s. | n.s. |  |
| CpG island count (linear term) | -2.9 | 4.4x10^-2^ | 5.0 | 0.08 |
| *CpG island count (quadratic term)* | 4.8 | < 10^-4^ | 2.8 |  |
| Poly(A/T)_n_ content (linear term) | 3.9 | 1.1x10^-3^ | 2.1 | 0.05 |
| *Poly(A/T)_n_ content (quadratic term)* | -3.7 | 3.1x10^-3^ | 2.1 |  |
| Gene content | n.s. | n.s. | n.s. | n.s. |
| LINE count | n.s. | n.s. | n.s. | n.s. |
| Multiple R^2^ |  |  |  | 0.11 |
| Adjusted R^2^ |  |  |  | 0.11 |

^a^*t* value – test statistic of null hypothesis that each predictor’s coefficient is equal to zero

^b^*P* values adjusted for multiple tests (using Bonferroni correction)

^c^VIF – variance inflation factor (see Materials and Methods)

^d^Relative contribution to explained variability computed for each predictor (see Materials and Methods)

^e^n.s. – non-significant (P >0.05)

The predictors are shown in the order of their relative contribution to explained variability for insertions in Table 1, except for the two recombination rates listed together.

B. Deletions

| Predictors | *t* value^a^ | Significance^b^ | VIF^c^ | Variability Explained^d^ |
| --- | --- | --- | --- | --- |
| X chromosome/autosome indicator | -20 | < 10^-4^ | 1.1 | 0.47 |
| Divergence | n.s.^e^ | n.s. | n.s. | n.s. |
| SINE count (linear term) | -10 | < 10^-4^ | 3.7 | 0.13 |
| *SINE count (quadratic term)* | 7.0 | < 10^-4^ | 2.0 |  |
| Distance to the telomere | n.s. | n.s. | n.s. | n.s. |
| Female recombination rate | n.s. | n.s. | n.s. | n.s. |
| Male recombination rate | 4.5 | < 10^-4^ | 1.4 | 0.03 |
| GC content | -3.6 | 3.5 x10^-3^ | 5.9 | 0.02 |
| CpG island count | n.s. | n.s. | n.s. | n.s. |
| Poly(A/T)_n_ content | n.s. | n.s. | n.s. | n.s. |
| Gene content | -6.0 | < 10^-4^ | 1.3 | 0.04 |
| LINE count | n.s. | n.s. | n.s. | n.s. |
| Poly(R/Y)_n__content | 4.1 | 4.5x10^-4^ | 1.4 | 0.02 |
| Multiple R^2^ |  |  |  | 0.24 |
| Adjusted R^2^ |  |  |  | 0.24 |

^a^*t* value – test statistic of null hypothesis that each predictor’s coefficient is equal to zero

^b^*P* values adjusted for multiple tests (using Bonferroni correction)

^c^VIF – variance inflation factor (see Materials and Methods)

^d^Relative contribution to explained variability computed for each predictor (see Materials and Methods)

^e^n.s. – non-significant (*P* >0.05)

The predictors are shown in the order of their relative contribution to explained variability for insertions in Table 1, except for the two recombination rates listed together.
