# Supplementary material for: A Macaque's-Eye View of Human Insertions and Deletions: Differences in Mechanisms
Source: PLoS Comput Biol. 2007 Sep 14;3(9):e176. doi: 10.1371/journal.pcbi.0030176 (PMC1976337; doi:10.1371/journal.pcbi.0030176)
Supplement: Supplementary file 9 [file pcbi.0030176.st005.doc]

A. Insertions

| Predictors | *t* value^a^ | Significance^b^ | VIF^c^ | Variability Explained^d^ |
| --- | --- | --- | --- | --- |
| Divergence (linear term) | 6.9 | < 10^-4^ | 2.6 | 0.12 |
| *Divergence (quadratic term)* | -8.3 | < 10^-4^ | 1.8 |  |
| SINE count | 8.2 | < 10^-4^ | 3.1 | 0.11 |
| Distance to the telomere (linear term) | -9.5 | < 10^-4^ | 2.0 | 0.15 |
| *Distance to the telomere (quadratic term)* | 6.3 | < 10^-4^ | 1.7 |  |
| Female recombination rate | 6.6 | < 10^-4^ | 1.3 | 0.07 |
| Male recombination rate | n.s.^e^ | n.s. | n.s. | n.s. |
| GC content (linear term) | -5.1 | < 10^-4^ | 3.5 | 0.04 |
| CpG island count (linear term) | n.s. | n.s. | n.s. | 0.04 |
| *CpG island count (quadratic term)* | 4.7 | < 10^-4^ | 3.3 |  |
| Poly(A/T)_n_ content (linear term) | 4.5 | < 10^-4^ | 2.4 | 0.03 |
| *Poly(A/T)_n_ content (quadratic term)* | -3.1 | 2.8 x10^-2^ | 2.1 |  |
| Gene content | 3.4 | 9.5 x10^-3^ | 1.3 | 0.02 |
| LINE count | n.s. | n.s. | n.s. | n.s. |
| Multiple R^2^ |  |  |  | 0.20 |
| Adjusted R^2^ |  |  |  | 0.20 |

^a^*t* value – test statistic of null hypothesis that each predictor’s coefficient is equal to zero

^b^*P* values adjusted for multiple tests (using Bonferroni correction)

^c^VIF – variance inflation factor (see Materials and Methods)

^d^Relative contribution to explained variability computed for each predictor (see Materials and Methods)

^e^n.s. – non-significant (*P* > 0.05)

The predictors are shown in the order of their relative contribution to explained variability for insertions in Table 1, except for the two recombination rates listed together.

B. Deletions

| Predictors | *t* value^a^ | Significance^b^ | VIF^c^ | Variability Explained^d^ |
| --- | --- | --- | --- | --- |
| Divergence (linear term) | 12 | < 10^-4^ | 2.5 | 0.23 |
| *Divergence (quadratic term)* | -8.9 | < 10^-4^ | 1.7 |  |
| SINE count (linear term) | -9.5 | < 10^-4^ | 4.0 | 0.15 |
| *SINE count (quadratic term)* | 3.6 | 3.6x10^-3^ | 2.4 |  |
| Distance to the telomere | -3.1 | 2.6 x10^-2^ | 1.3 | 0.01 |
| Female recombination rate | n.s.^e^ | n.s. | n.s. | n.s. |
| Male recombination rate | 4.5 | < 10^-4^ | 1.4 | 0.03 |
| GC content | -6.3 | < 10^-4^ | 6.1 | 0.06 |
| CpG island count | n.s. | n.s.. | n.s. | n.s. |
| Poly(A/T)_n_ content | 3.1 | 2.6 x10^-2^ | 1.1 | 0.01 |
| Gene content | n.s. | n.s. | n.s. | n.s. |
| LINE count | 5.3 | < 10^-4^ | 1.1 | 0.04 |
| Multiple R^2^ |  |  |  | 0.21 |
| Adjusted R^2^ |  |  |  | 0.21 |

^a^*t* value – test statistic of null hypothesis that each predictor’s coefficient is equal to zero

^b^*P* values adjusted for multiple tests (using Bonferroni correction)

^c^VIF – variance inflation factor (see Materials and Methods)

^d^Relative contribution to explained variability computed for each predictor (see Materials and Methods)

^e^n.s. – non-significant (P > 0.05)

The predictors are shown in the order of their relative contribution to explained variability for insertions in Table 1, except for the two recombination rates listed together.
