# Supplementary material for: A Macaque's-Eye View of Human Insertions and Deletions: Differences in Mechanisms
Source: PLoS Comput Biol. 2007 Sep 14;3(9):e176. doi: 10.1371/journal.pcbi.0030176 (PMC1976337; doi:10.1371/journal.pcbi.0030176)
Supplement: Supplementary file 10 [file pcbi.0030176.st006.doc]

| Predictors | Insertions | | | |  | | Deletions | | |
| --- | --- | --- | --- | --- | --- | --- | --- | --- | --- |
|  | 0.1 Mb | 0.5 Mb | 1 Mb | 10 Mb |  | 0.1 Mb | | 0.5 Mb | 1 Mb |
| X chromosome/autosome indicator | 0.06 | 0.07 | 0.08 | 0.05 |  | 0.3 | | 0.27 | 0.26 |
| Divergence | 0.12 | 0.1 | 0.09 | n.s. |  | 0.12 | | 0.2 | 0.15 |
| SINE count | 0.16 | 0.11 | 0.1 | n.s. |  | 0.04 | | 0.05 | 0.06 |
| Distance to the telomere | 0.08 | 0.06 | 0.05 | 0.03 |  | 0.06 | | 0.01 | 0.01 |
| Sex-averaged recombination rate | 0.03 | 0.08 |  |  |  | n.s. | | 0.01 |  |
| Female recombination rate |  |  | 0.01 | 0.03 |  |  | |  | n.s. |
| Male recombination rate |  |  | 0.04 | 0.04 |  |  | |  | 0.02 |
| GC content | 0.08 | 0.04 | 0.03 | n.s. |  | 0.11 | | 0.05 | 0.06 |
| CpG island count | 0.02 | 0.03 | 0.03 | 0.04 |  | n.s. | | 0.01 | n.s. |
| Gene content | 0.11 | 0.03 | 0.01 | n.s. |  | 0.04 | | n.s. | n.s. |
| Poly(A/T) content | 0.01 | 0.02 | 0.02 | n.s. |  | n.s. | | n.s. | n.s. |
| LINE count | n.s | n.s | n.s. | 0.08 |  | 0.02 | | 0.03 | 0.02 |
| Autosomes | 0.03 | 0.03 | 0.03 | 0.03 |  | 0.05 | | 0.02 | 0.02 |
| Multiple R^2^ | 0.07 | 0.19 | 0.27 | 0.62 |  | 0.07 | | 0.22 | 0.32 |
| Adjusted R^2^ | 0.06 | 0.18 | 0.27 | 0.6 |  | 0.07 | | 0.22 | 0.32 |
| Significant autosomes | 2, 3, 8, 18, 21 | 2, 3, 21 | 2, 21 | 21 |  | 4, 9, 16, 21 | | 16, 21 | 16, 21 |

^a^n.s. – non-significant
